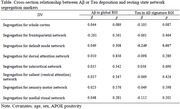# Association between brain tau deposition and default mode network connectivity in cognitively normal older adults

**DOI:** 10.1002/alz.090633

**Published:** 2025-01-09

**Authors:** Woo‐Jin Cha, Dahyun Yi, Evgeny J. Chumin, Min Soo Byun, Joon Hyung Jung, Hyejin Ahn, Yu Kyeong Kim, Yun‐Sang Lee, Koung Mi Kang, Chul‐Ho Sohn, Shannon L. Risacher, Olaf Sporns, Kwangsik Nho, Andrew J. Saykin, Dong Young Lee, KBASE Research Group

**Affiliations:** ^1^ Department of Neuropsychiatry, Seoul National University Hospital, Seoul Korea, Republic of (South); ^2^ Institute of Human Behavioral Medicine, Medical Research Center, Seoul National University, Seoul Korea, Republic of (South); ^3^ Center for Neuroimaging, Department of Radiology and Imaging Sciences, Indiana University School of Medicine, Indianapolis, IN USA; ^4^ Indiana Alzheimer’s Disease Research Center, Indianapolis, IN USA; ^5^ Department of Psychiatry, Seoul National University College of Medicine, Seoul Korea, Republic of (South); ^6^ Chungbuk National University Hospital, Cheongju Korea, Republic of (South); ^7^ Interdisciplinary program of cognitive science, Seoul National University, Seoul Korea, Republic of (South); ^8^ Department of Nuclear Medicine, SMG‐SNU Boramae Medical Center, Seoul Korea, Republic of (South); ^9^ Department of Nuclear Medicine, Seoul National University College of Medicine, Seoul Korea, Republic of (South); ^10^ Department of Radiology, Seoul National University Hospital, Seoul Korea, Republic of (South); ^11^ Department of Psychological and Brain Sciences, Indiana University, Bloomington, IN USA; ^12^ Indiana Alzheimer’s Disease Research Center, Indiana University School of Medicine, Indianapolis, IN USA; ^13^ Center for Computational Biology and Bioinformatics, Indiana University School of Medicine, Indianapolis, IN USA; ^14^ Indiana Alzheimer's Disease Research Center, Indianapolis, IN USA; ^15^ Department of Medical and Molecular Genetics, Indiana University School of Medicine, Indianapolis, IN USA

## Abstract

**Background:**

Alzheimer’s disease (AD) pathology occurs in the brain before manifestation of significant cognitive decline. Growing evidence suggests that brain networks such as default mode network (DMN) or salience network, identified through resting‐state functional magnetic resonance imaging (MRI), are affected by AD pathology. In this study, we investigated the relationship between network segregation and the key in vivo AD pathologies including beta‐amyloid (Aβ) and tau deposition in old adults with no cognitive impairment.

**Method:**

A total 283 older adults with normal cognition aging from 55 to 87 were recruited from the Korean Brain Aging Study for the Early Diagnosis and Prediction of Alzheimer’s Disease (KBASE) cohort. The participants underwent comprehensive clinical and neuropsychological assessment, [^11^C] Pittsburgh Compound B PET for measuring Aβ deposition, [^18^F] AV‐1451 PET for measuring tau deposition, structural MRI, and resting‐state functional MRI for measuring functional connectivity (FC). For PET scans, standard uptake value ratio (SUVR) was used for the analyses; combined regions of inferior cerebellum and pons were used as the reference region when obtaining SUVRs. For FC, segregation values (ratios between median z‐transformed Pearson correlation of within‐ and between‐network connectivity) for overall and the seven individual resting state networks were computed (Table). The relationships between Aβ or tau deposition and network connectivity segregation were examined through cross‐sectional approach using multiple regression analyses. In the analyses, Aβ or tau deposition was used as an independent variable and segregation values of the networks were used as dependent variables.

**Result:**

Tau deposition had a significant negative association with the DMN segregation (β = ‐0.249, p = 0.007); but, tau had no relationships with any other networks (Table). Aβ deposition was not associated with any segregation values for the seven brain networks (Table).

**Conclusion:**

Our finding suggests that impaired functional connectivity of DMN is closely linked to tau deposition even in cognitively unimpaired older individuals.